# The Impact of Slice Interval and Equation on the Accuracy of Magnetic Resonance Image Estimation of Quadriceps Muscle Volume in End Stage Liver Disease

**DOI:** 10.3389/fresc.2022.854041

**Published:** 2022-04-06

**Authors:** Jonathan I. Quinlan, Clare Jones, Emma Bissonnette, Amritpal Dhaliwal, Felicity Williams, Surabhi Choudhary, Leigh Breen, Gareth G. Lavery, Matthew J. Armstrong, Ahmed M. Elsharkawy, Janet M. Lord, Carolyn A. Greig

**Affiliations:** ^1^NIHR Birmingham Biomedical Research Centre, University Hospitals Birmingham NHS Foundation Trust and University of Birmingham, Birmingham, United Kingdom; ^2^School of Sport, Exercise and Rehabilitation Sciences, University of Birmingham, Birmingham, United Kingdom; ^3^Institute of Inflammation and Ageing, University of Birmingham, Birmingham, United Kingdom; ^4^Therapies Department, University Hospitals Birmingham, Birmingham, United Kingdom; ^5^Department of Imaging, University Hospitals Birmingham, Birmingham, United Kingdom; ^6^MRC-Versus Arthritis Centre for Musculoskeletal Ageing Research, University of Birmingham, Birmingham, United Kingdom; ^7^Department of Biosciences, Nottingham Trent University, Nottingham, United Kingdom; ^8^Liver Unit, Queen Elizabeth Hospital Birmingham, Birmingham, United Kingdom

**Keywords:** MRI, muscle volume, end stage liver disease, muscle mass, sarcopenia

## Abstract

**Introduction:**

End stage liver disease (ESLD) is associated with loss of muscle mass and function, known as sarcopenia, which can increase the risk of complications of ESLD, hospitalization and mortality. Therefore, the accurate assessment of muscle mass is essential to evaluate sarcopenia in ESLD. However, manual segmentation of muscle volume (MV) can be laborious on cross-sectional imaging, due to the number of slices that require analysis. This study aimed to investigate the impact of reducing the number of slices required for MV estimation. Further, we aimed to compare two equations utilized in estimating MV (cylindrical and truncated cone).

**Methods:**

Thirty eight ESLD patients (23 males; 54.8 ± 10.7 years) were recruited from the Queen Elizabeth University Hospital Birmingham. A 3T MRI scan was completed of the lower limbs. Quadriceps MV was estimated utilizing 1-, 2-, 3-, and 4 cm slice intervals with both cylindrical and truncated cone equations. Absolute and relative error (compared to 1 cm slice interval) was generated for 2-, 3-, and 4 cm slice intervals. L3 skeletal muscle index (SMI) was also calculated in 30 patients.

**Results:**

Relative error increased with slice interval using the cylindrical (0.45 vs. 1.06 vs. 1.72%) and truncated cone equation (0.27 vs. 0.58 vs. 0.74%) for 2, 3, and 4 cm, respectively. Significantly, the cylindrical equation produced approximately twice the error compared to truncated cone, with 3 cm (0.58 vs. 1.06%, *P* < 0.01) and 4 cm intervals (0.74 vs. 1.72%, *P* < 0.001). Finally, quadriceps MV was significantly correlated to L3 SMI (*r*^2^ = 0.44, *P* < 0.0001).

**Conclusion:**

The use of the truncated equation with a 4 cm slice interval on MRI offers an efficient but accurate estimation of quadricep muscle volume in ESLD patients.

## Introduction

End stage liver disease (ESLD) is the leading cause of death among adults aged 35–49 years in the UK and mortality rate has increased 4-fold since 1970 (1). A common and significant complication of ESLD is sarcopenia, in which muscle mass, quality and function become compromised. It is believed that sarcopenia affects ~22–70% of ESLD patients (2) and can have a significant impact upon patient quality of life, risk of mortality and may adversely affect the outcome of liver transplantation (3). Thus, the accurate and reliable assessment of muscle mass in ESLD is vital to assist in the identification and treatment of sarcopenia.

At present, there is a lack of comprehensive research into the assessment of sarcopenia in relation to ESLD, although a large range of sarcopenia prevalence has been reported. This is partly due to discordance in specific clinical definitions and applicable diagnostic cut-off values (4). Currently, the gold standard method of assessing sarcopenia in ESLD is based on anatomical cross-sectional area (ACSA) of a single axial computed tomography (CT) scan, typically at the third lumbar vertebral level (L3). Indeed, significant attempts have been made to develop these cut-off values for L3 skeletal muscle index (SMI) (5). Specific cut offs for L3 SMI of <50 and <39 cm^2^/m^2^ have previously been developed in a large multi-center study for men and women, respectively (3). However, as there is an associated radiation risk involved with CT scans it would be shrewd to implement alternative methodologies to assess and monitor sarcopenia status. One such method is magnetic resonance imaging (MRI) which offers excellent image resolution for the analysis of muscle mass, compared with CT. Although the assessment of L3 SMI is common, it has been suggested that the quadricep muscles may offer a more suitable alterative for the assessment of muscle mass (6); primarily due to clear defined muscle borders and a larger sensitivity to change in disease states associated with sarcopenia (7, 8). Indeed, the lower extremities contain >50% of total body muscle mass, of which a large majority is contained within the thigh (9). In addition, the quadricep muscles are known to correlate with functional ability, unlike muscles at L3 that are minimally influenced by activity levels (6, 10). It is for this reason that quadriceps muscle volume (MV) is widely used as a measure of muscle mass in age-related sarcopenia research as well as in targeted intervention studies (11–13). However, to the best of our knowledge, quadriceps MV has not previously been evaluated in an ESLD population.

The calculation of quadriceps MV typically requires the manual segmentation of multiple MRI ACSA slices, such that the summation of volumes between slices equates to total MV. Whilst manual segmentation is very time intensive and challenging to implement in large research studies/clinical practice, it is still considered the most accurate method of quantifying skeletal MV (14). To overcome the time consuming nature of manual segmentation, several studies have investigated the impact of increasing the slice interval (and hence reduce the number of slices required) on MV accuracy (7, 15–17). However, these studies have all been completed in healthy populations and thus it is currently unknown whether the same time saving approaches can be applied to ESLD.

Aside from the impact of slice interval on the accuracy of MV estimations, investigators are typically required to utilize mathematical equations to calculate MV. The two most widely used methods are the cylindrical (or Cavalieri) equation and the truncated cone equation. Previous studies are somewhat inconclusive as to which is the most appropriate to use; however there is a suggestion that the cylindrical equation is the more accurate of the two (15, 17, 18). Nonetheless, it is unclear how these equations would influence MV estimations and subsequent error in a challenging ESLD cohort.

Thus, the aim of this study was 2-fold. Firstly, to assess the impact of slice interval on the accuracy of quadriceps MV estimation in a cohort of ESLD patients. And secondly, to ascertain whether cylindrical or truncated cone equations produce less error in the estimation of quadriceps MV when slice interval is increased. We hypothesize that a larger slice interval would result in higher absolute and relative error and, consistent with previous work in healthy individuals, the cylindrical equation would result in smaller errors.

## Materials and Methods

### Participants

We recruited 38 ESLD patients from the Queen Elizabeth Hospital (Birmingham, UK) liver transplant waiting list clinic (23m/15f) as part of a larger, prospective observational study (ClinicalTrials.gov Identifier: NCT04734496). Patients had a mean age of 54.8 ± 10.7 years and a mean BMI of 29.8 ± 6.7 kg/m^2^. Disease etiology consisted of alcohol related liver disease (*n* = 18), primary sclerosing cholangitis (*n* = 9), primary biliary cirrhosis (*n* = 4), non-alcoholic liver disease (*n* = 5) and other (*n* = 2). Disease severity was determined by a mean Model for End Stage Liver Disease (MELD) score of 13.7 ± 4.7 and 71% had either Childs-Pugh B/C. The study was approved by the Health Research Authority - West Midlands Solihull Research Ethics Service Committee Authority (REC reference: 18/WM/0167). All patients provided written informed consent.

### MRI Protocol and Quadriceps Manual Segmentation

**Figure 1:**
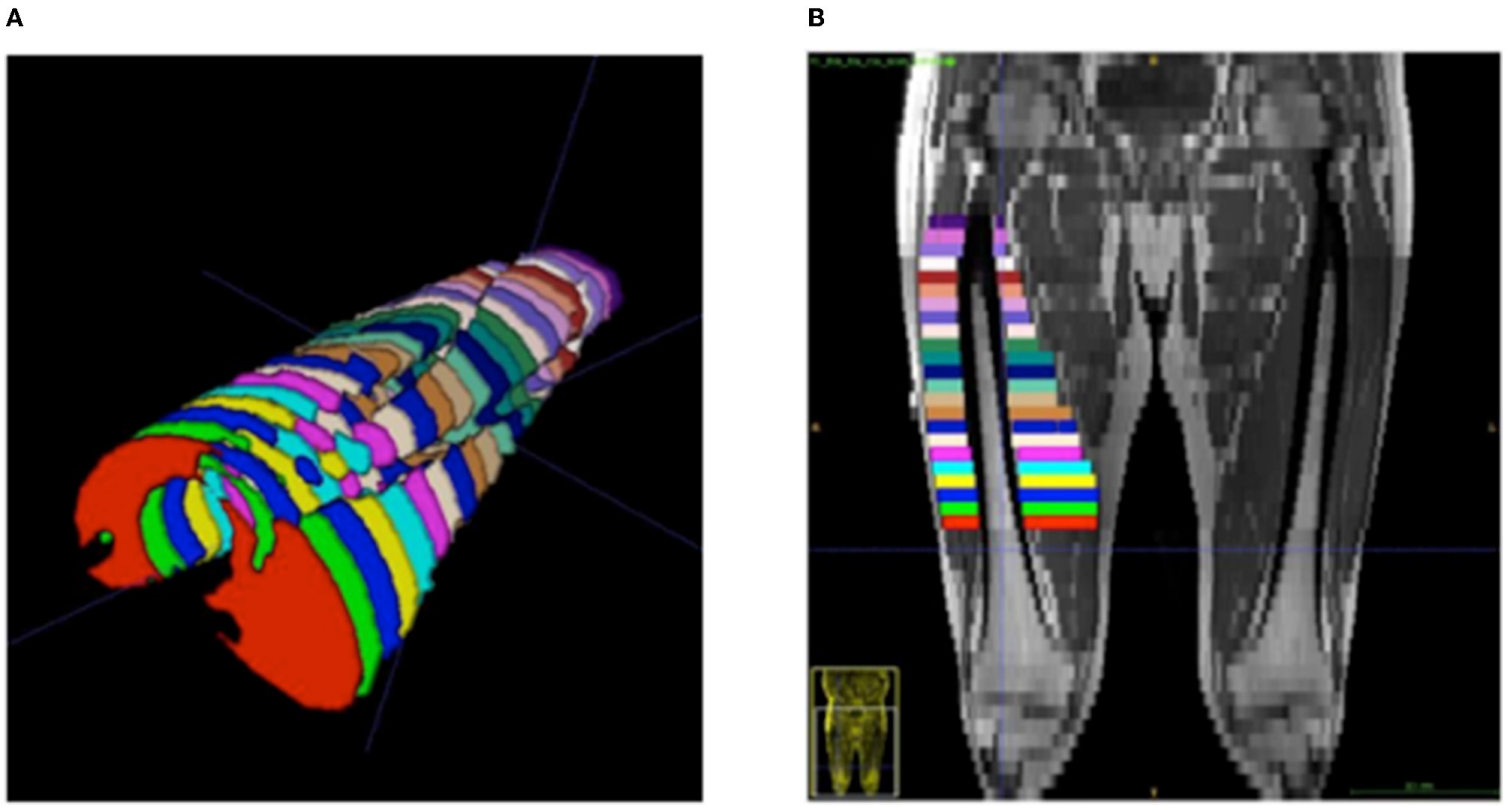
Representative image of a segmented quadricep femoris muscle from an ESLD patient. **(A)** Viewing in a cephalad direction, each color represents a separate cross section at 1 cm inter-slice distance, **(B)** a coronal plane MRI demonstrating the distribution and location of equivalent segments.

Images were collected using a 3T Cobalt MRI scanner with a T1 VIBE protocol: repetition time 600 ms, echo time 15.2 ms, field of view 512 × 512 mm, slice interval 1 cm, with no gap between slices. Offline manual segmentation of the quadriceps muscle group from the dominant leg was completed by two investigators (EB, CJ) via ITK-SNAP (version 3.8.0). For the calculation of MV, ~23 ACSA slices were analyzed across a restricted quadriceps region of interest (ROI) (Figure 1). Similar to previous work, both proximal and distal extremes of the quadriceps were omitted from analysis to increase accuracy (19). The proximal limit was identified as the appearance of the lesser trochanter and the distal limit as 20% above the proximal aspect of the patella (Figure 1). For individual ACSA analysis, any sections which were distinctively identified as inter or intramuscular adipose tissue or non-contractile tissue were not included in muscle ACSA analysis. Following the assessment of muscle ACSA, MV was estimated via two previously utilized equations; cylindrical (Equation 1) and truncated cone (Equation 2). For each equation, MV was calculated utilizing slice intervals of 1, 2, 3, and 4 cm.


$$\begin{array}{l} MV_{cylinder}=\;\Sigma \left(CSA_{n}\;\times \;slice\;distance\right) \end{array}$$

Equation 1: Cylindrical calculation of muscle volume.


$$\begin{array}{l} \;MV_{cone}=\Sigma \left((\frac{1}{3}\times slice\;thickness\right)\times \\ \left[CSA_{n}+\sqrt{\left(CSA_{n}\times CSA_{n+1}\right)}+CSA_{n+1}\right]) \end{array}$$

Equation 2: Truncated cone calculation of muscle volume, whereby both the current and sequential ACSA are required for the estimation.

### L3 SMI Estimation

Muscle CSA at the L3 level was manual segmented via Horos software (version 3.3.6) by a single investigator (JQ). Muscles at the L3 region included the psoas, erector spinae, quadratus lumborum, transversus abdominus, external and internal obliques, and rectus abdominus. Consequently, L3 SMI was calculated by dividing L3 muscle CSA by the squared height of each patient (cm^2^/m^2^). This analysis was only available for *n* = 30.

### Muscle Volume Inter-rater Reliability

Despite moderate to excellent inter-rater reliability for muscle manual segmentation being reported elsewhere [19]; we wanted to test the reliability within an ESLD population. Therefore, a separate experienced investigator (JQ) randomly selected eight scans within the study for the inter-rater reliability to be assessed via intraclass correlation analysis (ICC), completed using SPSS (IBM corp. version 26.0). The results of the two-way mixed ICC analysis were as follows: within the cylindrical equation = 0.999 (95% confidence = 0.989–1.00), and with the truncated cone equation ICC = 0.999 (95% confidence interval = 0.95–1.00).

### Statistical Analysis

To compare the impact of slice interval for the assessment of MV, Bland-Altman plots were created via GraphPad Prism (Version 9.2.0) to determine mean bias and 95% limits of agreement (LOA) (20). The comparator method of MV estimation (i.e., 1 cm slice interval) for both equations were compared against 2, 3, and 4 cm slice intervals and the difference plotted against average volume measures [i.e., (A – B) vs. average, whereby A is 1 cm slice interval and B is 2, 3, or 4 cm]. In addition, the absolute (cm^3^) and relative (%) error for MV was calculated for 2-, 3-, and 4 cm slice intervals. Specifically, this was calculated as the difference in MV estimation compared to MV value obtained with 1 cm slice interval. To compare the effect of increased slice interval as well as the effect of equation on MV estimations, a two-way ANOVA with Tukey *post-hoc* analysis was completed. Finally, Pearson's *r* was used to assess for correlation between muscle volume and L3 SMI. For all tests, results were considered statistically significant when *P* < 0.05.

## Results

### Slice Interval

**Figure 2:**
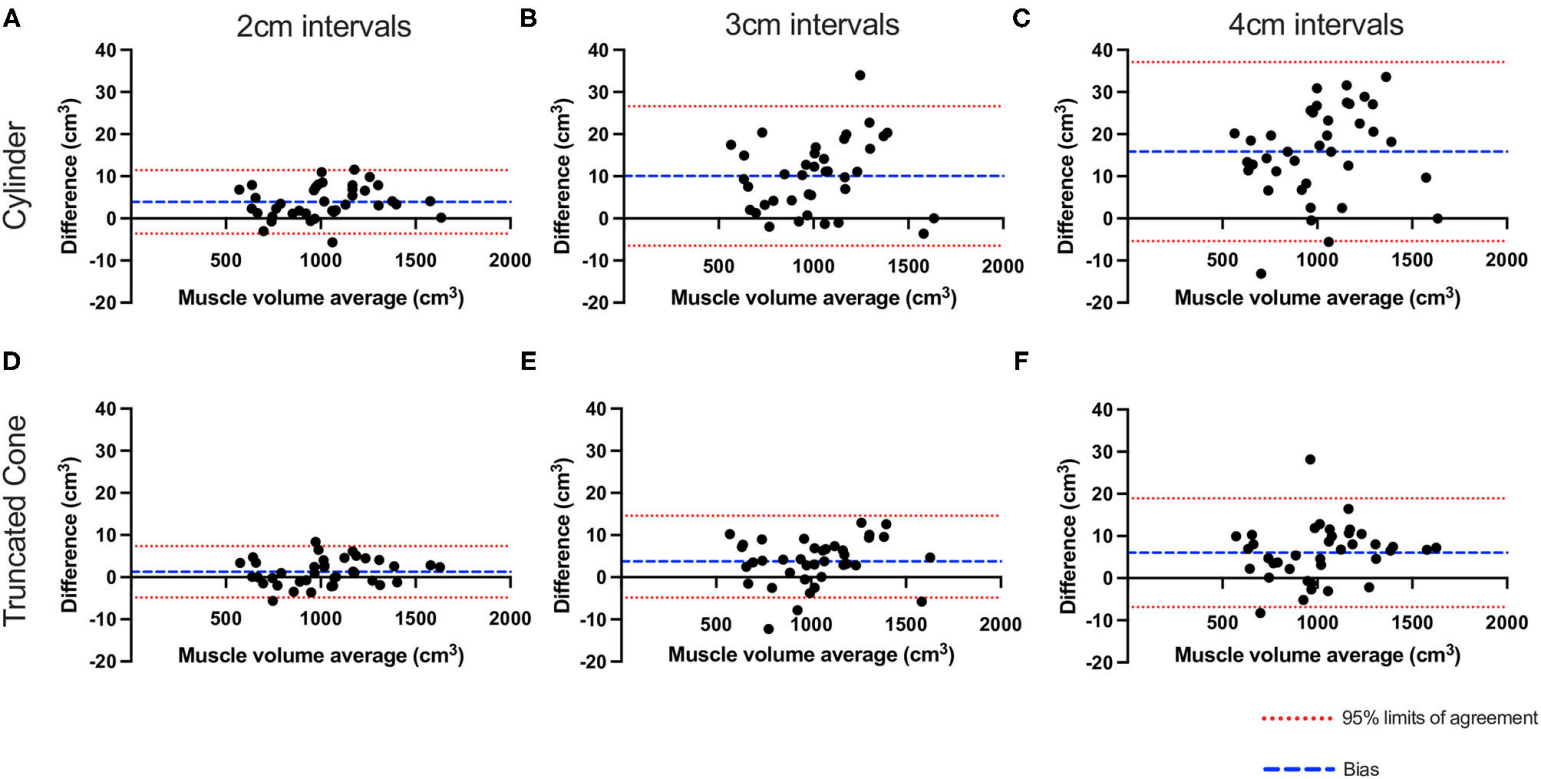
Bland–Altman plots showing the effect of slice interval on quadriceps muscle volume estimation. Plotted values show the difference between 1 cm slice interval and values generated from 2- **(A,D)**, 3- **(B,E)**, and 4 cm slice interval **(C,F)** utilizing the cylinder **(A–C)** and truncated cone equations **(D–F)**. Red dashed line represents 95% LOA and blue dash represents the mean bias.

**Figure 3:**
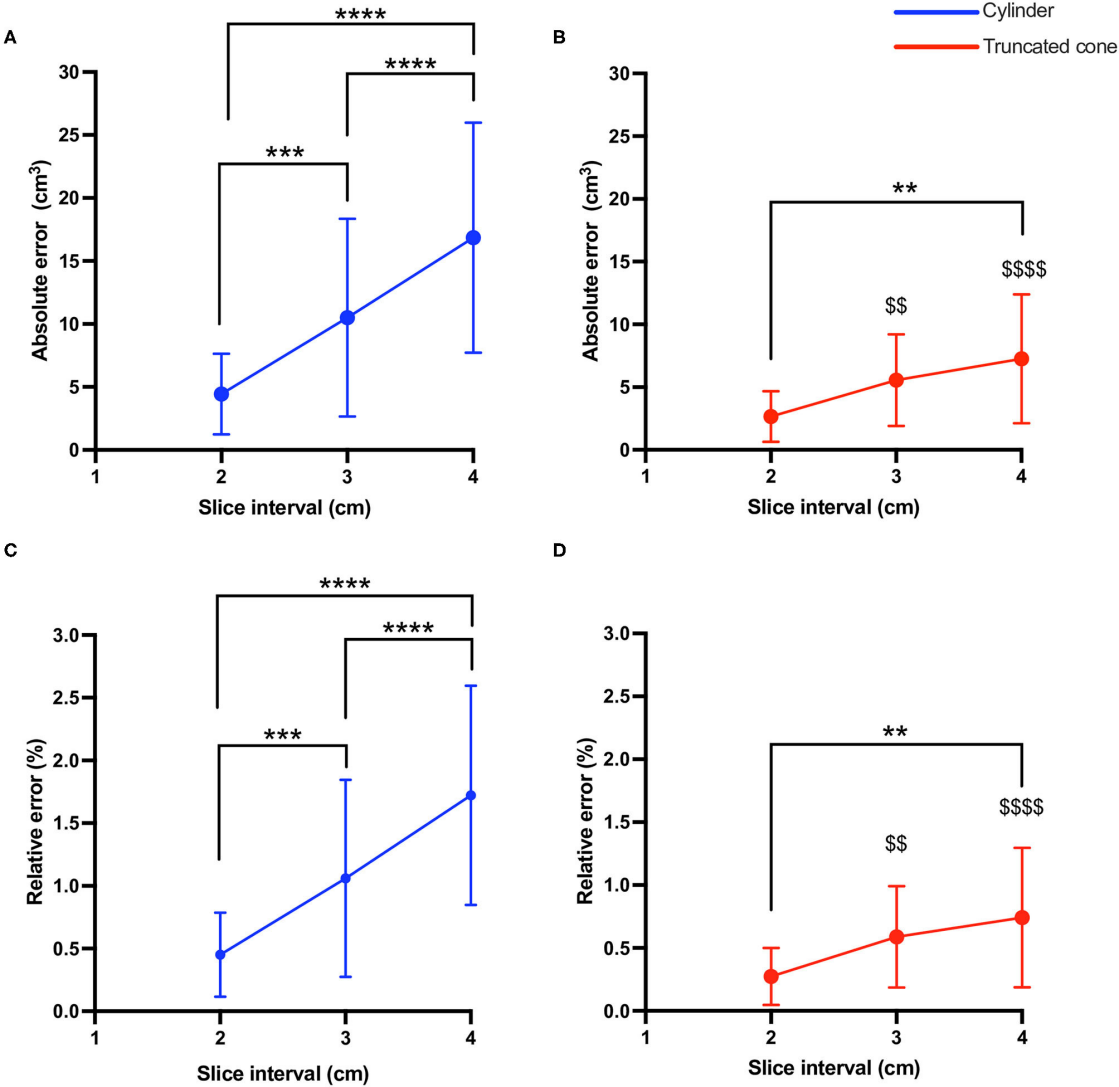
Absolute **(A,B)** and relative **(C,D)** error with increased slice interval compared with 1 cm slice intervals using cylinder (blue) and truncated cone equation (red). *P*-values for slice interval comparison within equation were **P < 0.01, ***P < 0.001, and ****P < 0.0001. *P*-values for comparing either absolute error or relative error between equations (at same slice interval) were $$ *P* < 0.01, $$$$ *P* < 0.0001.

Bland-Altman analysis (Figure 2) revealed that almost all data points fell within the 95% LOA with 2 cm (36/38, 95% and 36/38, 95%) 3 cm (37/38, 97% and 36/38 95%) and 4 cm slice intervals (36/38, 95% and 35/38, 92%) for cylinder and truncated cone, respectively. The plots demonstrated that as the slice interval increased, the agreement for the 1 cm slice interval decreased. Both cylinder and truncated cone equations demonstrated a positive mean bias (i.e., underestimation) at all slice intervals. Mean bias values (95% LOA) for the cylindrical equation were +3.9 cm^3^ (−3.6 to 11.4), +10.0 cm^3^ (−6.4 to 26.6 cm^3^) and +15.8 cm^3^ (−5.4 to 37.0 cm^3^) for 2, 3, and 4 cm, respectively. The truncated cone equation demonstrated consistently lower mean bias and LOA values: +1.2 cm^3^ (−4.8 to 7.3 cm^3^), +3.7 cm^3^ (−7.0 to 14.5 cm^3^) and +6.0 cm^3^ (−6.8 to 18.9 cm^3^) for 2, 3, and 4 cm, respectively. Absolute error increased significantly in cylinder equation between 2 and 3 cm (4.4 ± 3.1 cm^3^ vs. 10.5 ± 7.8 cm^3^ *P* < 0.001), 2 and 4 cm (4.4 ± 3.1 cm^3^ vs. 16.850 cm^3^, *P* < 0.0001) and from 3 to 4 cm (10.5 ± 7.8 cm^3^ vs. 16.9 cm^3^ *P* < 0.0001) (Figure 3A). Whereas, an increased error was only seen between 2 and 4 cm for the truncated cone equation (2.6 ± 2.0 cm^3^ vs. 7.2 ± 5.1 cm^3^ *P* < 0.01, Figure 3B). Relative error (%) also significantly increased using the cylinder equation between 2 and 3 cm (0.45 ± 0.3% vs. 1.06 ± 0.7% *P* < 0.001), 2 and 4 cm (0.45 ± 0.3% vs. 1.72 ± 0.8% *P* < 0.0001), and 3 and 4 cm (1.06 ± 0.7% vs. 1.72 ± 0.8% *P* < 0.0001) (Figure 3C). Relative error using the truncated cone equation increased only between 2 and 4 cm intervals (0.27 ± 0.2%, vs. 0.74 ± 0.5%, *P* < 0.01), and was unchanged between 2 and 3 cm (0.27 ± 0.2% vs. 0.58 ± 0.4%) and 3 and 4 cm (0.58 ± 0.4% vs. 0.74 ± 0.5%) (Figure 3D).

### Cylinder vs. Truncated Cone Estimates

2-way ANOVA analysis showed a significant interaction (*P* = 0.0001) and main effect for slice interval (*P* < 0.0001) and equation (*P* < 0.0001). Tukey's *post-hoc* multiple comparisons showed that the truncated cone equation produced significantly less absolute error at 3 cm (5.5 ± 3.6 cm^3^ vs. 10.5 ± 7.8 cm^3^ P < 0.001) and 4 cm intervals (7.2 ± 5.1 cm^3^ vs. 16.8 ± 9.1 cm^3^ *P* < 0.0001). Similarly, relative error was also lower using the truncated cone equation compared with cylinder at both 3 cm (0.58 ± 0.4% vs. 1.0 ± 0.8% *P* < 0.001) and 4 cm intervals (0.74 ± 0.5% vs. 1.7 ± 0.9% *P* < 0.0001).

### Correlation Between L3 SMI and Muscle Volume

**Figure 4:**
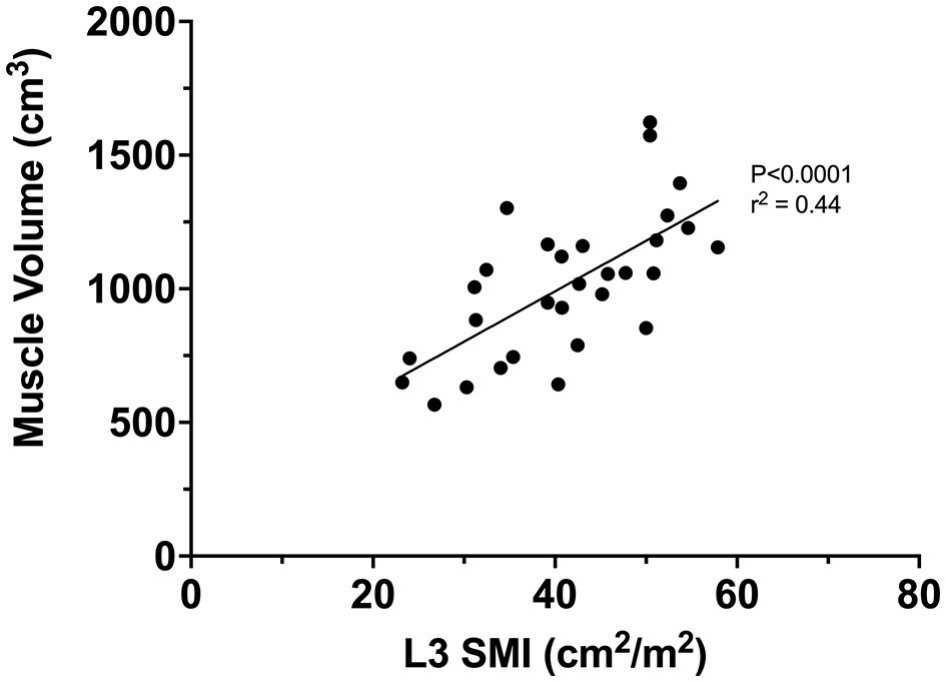
Correlation between L3 SMI and quadriceps muscle volume (estimated via truncated cone and 4 cm interval).

A significant positive correlation was seen between quadriceps MV (estimated by truncated cone equation and 4 cm interval) and L3 SMI (*r*^2^ = 0.44, *P* < 0.0001, Figure 4).

## Discussion

The accurate estimation of MV is essential for understanding and evaluating sarcopenia in chronic disease states such as ESLD. However, the manual segmentation of muscle from MRI sequences is notoriously time consuming and open to interpretation error. Our data suggest that increasing the interslice distance from 1 cm to 2-, 3, and 4-cm, is associated with higher absolute and relative error. However, the relative error remained low at <1% for truncated cone and <2% for cylindrical for all inter-slice distances, even when increased to 4 cm. Bland-Altman analysis demonstrated that ~95% of the data points at 4 cm slice intervals fell within the 95% LOA when compared to 1 cm slice interval (37/38 points and 36/38 points for cylinder and truncated cone, respectively, within the 95% LOA). Contrary to our secondary hypothesis, our data suggest that the truncated cone equation produces less absolute and relative error than the cylindrical equation when using a restricted quadriceps muscle ROI in patients with ESLD. Finally, we also demonstrated a significant positive correlation between MV and the gold standard in the hepatology field, L3 SMI, highlighting the relevance of quadriceps MV.

### Inter-slice Distance

In agreement with our hypothesis, as slice interval increased from 1 to 4 cm, the error in MV estimation also increased, irrespective of the equation used. Although this observation was an expected outcome of our study when calculating volume from ACSA measurements alone (21), it is possible to estimate MV from a single ACSA slice (22). Nonetheless, whilst we observed an increase in both the relative and absolute error for both equations across the inter-slice distances (Figure 3), in agreement with previous research on healthy adults (16, 17), even at a slice interval of 4 cm, the relative error remained minimal (0.74 and 1.72% for truncated cone and cylinder, respectively). The measured error was likely caused by a systematic under-estimation of MV following the increase in slice interval as demonstrated via a positive bias in all cases (Figure 2). It is also possible that under-estimation bias was the result of an increased slice interval due to the irregularity of muscle distribution and quadriceps composition present in ESLD patients. Indeed, ESLD is associated with an increase in adipose tissue and particularly an infiltration of fat (IMAT) within the muscle (23). Inherently, an increase in IMAT makes manual segmentation of the quadriceps significantly more challenging and when the slice interval is increased, it may impose greater assumptions on distinguishing between muscle mass and other tissue. Indeed, if the analyzed slice is low in IMAT, then a larger slice interval assumes IMAT remains low (and muscle ACSA high) over this extended portion of the muscle. In contrast, if the analyzed slice is high in IMAT (and muscle ACSA low), then the reverse assumption would be made, i.e., lower muscle mass over a larger proportion. We observed an apparent under estimation following increased slice interval and thus the second assumption is likely true in this scenario. In addition to the IMAT artifact, when the slice interval is increased, it becomes increasingly more likely that an investigator may miss true peaks in muscle ACSA. In turn, the omission of these larger ACSA values from volume analysis may result in an underestimation of MV (16).

### Cylinder vs. Truncated Cone Equation

The secondary aim of this study was to investigate the impact of the mathematical equation (i.e., Cylinder vs. Truncate cone) on the estimation of MV in ESLD patients. Contrary to our hypothesis, we found that the truncated cone geometric model appeared more reliable (i.e., lower absolute and relative error across all slice distances) at estimating MV, compared with the cylindrical model. This was supported by consistently smaller 95% LOA, mean bias values, and both absolute and relative error (Figures 2, 3). Specifically, once the slice interval was increased beyond 2 cm, both the absolute and relative error were statistically higher with the cylindrical equation compared with truncated cone. Our data provide evidence for the use of the truncated cone equation in an ESLD population for the estimation of quadriceps MV.

**Figure 5:**
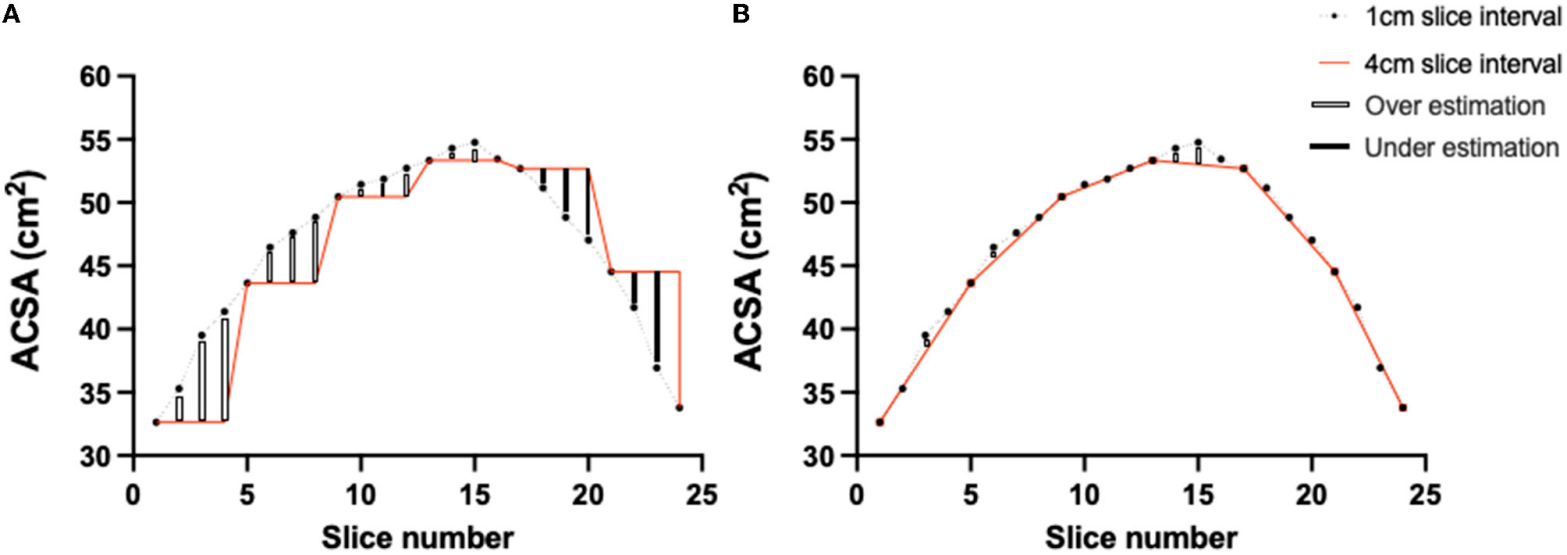
Representative schematic of ACSA obtained over the restricted ROI utilizing the cylindrical **(A)** and truncated cone equation **(B)** to demonstrate theoretical explanation for differences in error production. Dashed black line represents ACSA values obtained via 1 cm slice interval and red solid line at 4 cm slice interval. Open bars represent underestimation and solid bars overestimation of the 4 cm slice intervals when compared to 1 cm slice intervals.

The mathematical basis of the cylindrical equation assumes the measured ACSA is entirely unchanged for “x” distance and as such, produces both under and over estimations. Theoretically, these over- and underestimations would approximately equate to one another, and the errors would cancel each other out. By comparison, the truncated cone equation requires two ACSA values (i.e., ACSA value of slice 1 and the subsequent ACSA value with “x” slice interval); whereby the equation accounts for the change in size between two ACSA slices. Previous research has appeared inconclusive, but with a marginal lean toward the cylindrical equation for accurately calculating quadriceps MV (7, 17). Indeed, several authors have previously opposed the use of the truncated cone equation for MV estimations (7, 15, 17). It is pertinent to note that others have provided evidence to defend the use of the truncated cone equation (16). Interestingly, while Barnouin et al. (17) concluded that the truncated cone formula was the least accurate of the four methods they investigated (i.e., cylinder, truncated cone and two polynomial equations); they observed similar relative error between all methods up until 2.5 cm slice intervals. Further Tracy et al. (16) demonstrated an ~99% accuracy of the truncated cone equation when using 4 cm slice intervals (16). However, one important consideration between the above studies and our own, is the ROI for quadriceps MV estimation. The above studies incorporated full quadriceps MV (i.e., origin to insertion), whereas the methodology herein excludes the proximal and distal extremes (i.e., where ACSA changes between sequential slices are often large). Typically, it has been suggested that the convex shape of quadriceps prevents the truncated cone method from accurately estimating MV and in turn resulting in an overall underestimation (17). However, utilizing a restricted ROI likely removed regions where the truncated cone would produce high levels of underestimation error. Indeed, changes between sequential ACSAs in the remaining ROI are typically smaller and hence the truncated cone equation may prove more accurate. In contrast, the cylindrical approach does not perform as well within this restricted ROI. In full MV estimations, the cylindrical approach succeeds by theoretically canceling out under and over estimations. However, in the case of a restricted ROI, an unequal balance of under and over estimations exists, which would result in overall error (Figure 5). As such, if the cylindrical estimation of MV begins with distal ACSAs and works proximally (as herein), the cylindrical equation would underestimate MV as a greater proportion of underestimations would occur (Figure 5).

Aside from the mathematical rationale as to why our conclusions differ to those previously discussed, it is also worth noting that the above studies investigated healthy, non-diseased individuals. By contrast, our study specifically considered an ESLD population and so it may be difficult to directly compare outcomes. Indeed, it is likely that the accuracy of MV estimations may have been exacerbated by intrinsic factors associated with ESLD, such as myosteatosis. Aside from ESLD related influences, endogenous factors such as genetics and exogenous lifestyle factors, including reduced physical activity and/or poor nutritional intake may have collectively negatively influenced muscle volume and hence reduced the accuracy of our estimations (24–26). As mentioned previously, the cylindrical model heavily relies on the assumption of evenly distributed muscle mass throughout the limb (27). However, it has been shown that in clinical cohorts, the quadriceps display significant irregularities along the muscle length when compared with healthy individuals (28). Furthermore, it is likely that loss of muscle mass does not occur uniformly (29, 30), which could further compound the irregularity of muscle distribution and hence impact MV estimations via the cylindrical equation (28). In addition to changes in the quantity of muscle, there are also likely adverse changes to muscle quality, such as the infiltration of fat. Indeed, a higher prevalence of IMAT could further augment the irregular distribution of muscle tissue; thus, impacting the cylindrical equation more so than the truncated cone due the fundamental assumptions associated with the former.

## Conclusion

This is the first study to investigate the effects of slice interval and mathematical equation (i.e., cylindrical, and truncated cone) on MV estimations in patients with ESLD. We provide evidence for the preferential use of the truncated cone equation over the cylindrical equation when utilizing a restricted ROI approach such as herein. Further we demonstrate that the slice interval can be increased up to 4 cm whilst maintaining relatively low levels of absolute and relative error (7.2 cm^3^ and ~0.7% for truncated cone). Finally, we also show that quadriceps MV is significantly correlated to L3 SMI, demonstrating the relevance of our approach. These findings provide clear evidence and rationale for future investigators to utilize the truncated cone equation with larger interslice distances and in turn save considerable time in image processing and ultimately create a more feasible process of MV estimation.

## Data Availability Statement

The raw data supporting the conclusions of this article will be made available by the authors, without undue reservation.

## Ethics Statement

The studies involving human participants were reviewed and approved by Health Research Authority - West Midlands Solihull Research Ethics Service Committee Authority (REC reference: 18/WM/0167). The patients/participants provided their written informed consent to participate in this study.

## Author Contributions

JQ, CJ, and EB were all involved in acquiring and completing analysis of the data contained within. JQ constructed the initial draft of the manuscript. All authors aided in either the production or application of the protocol discussed within the manuscript and edited and approved the final version.

## Funding

This work was supported by the National Institute for Health Research Birmingham Biomedical Research Centre (BRC-1215-20009).

## Author Disclaimer

This paper presents independent research funded by the NIHR Birmingham Biomedical Research Centre at the University Hospitals Birmingham NHS Foundation Trust and the University of Birmingham. The views expressed are those of the author(s) and not necessarily those of the NHS, the NIHR or the Department of Health and Social Care.

## Conflict of Interest

The authors declare that the research was conducted in the absence of any commercial or financial relationships that could be construed as a potential conflict of interest.

## Publisher's Note

All claims expressed in this article are solely those of the authors and do not necessarily represent those of their affiliated organizations, or those of the publisher, the editors and the reviewers. Any product that may be evaluated in this article, or claim that may be made by its manufacturer, is not guaranteed or endorsed by the publisher.

## References

[1] Office for National Statistics. Liver Disease: Applying All Our Health. Available online at: https://www.gov.uk/government/publications/liver-disease-applying-all-our-health/liver-disease-applying-all-our-health#fn:2 (accessed September 14, 2021).

[2] Kim G Kang SH Kim MY Baik SK. Prognostic value of sarcopenia in patients with liver cirrhosis: a systematic review and meta-analysis. PLoS ONE. (2017) 12:1–16. 10.1371/journal.pone.0186990 29065187 PMC5655454

[3] Carey EJ Lai JC Wang CW Dasarathy S Lobach I Montano-Loza AJ. A multicenter study to define sarcopenia in patients with end-stage liver disease. Liver Transplant. (2017) 23:625–33. 10.1002/lt.24750 28240805 PMC5762612

[4] Sinclair M. Controversies in diagnosing sarcopenia in cirrhosis—Moving from research to clinical practice. Nutrients. (2019) 11:1–16. 10.3390/nu11102454 31615103 PMC6836123

[5] van Vugt JLA Levolger S de Bruin RWF van Rosmalen J Metselaar HJ IJzermans JNM. Systematic review and meta-analysis of the impact of computed tomography–assessed skeletal muscle mass on outcome in patients awaiting or undergoing liver transplantation. Am J Transplant. (2016) 16:2277–92. 10.1111/ajt.13732 26813115

[6] So JL Janssen I Heymsfield SB Ross R. Relation between whole-body and regional measures of human skeletal muscle. Am J Clin Nutr. (2004) 80:1215–21. 10.1093/ajcn/80.5.1215 15531668

[7] Nordez A Jolivet E Südhoff I Bonneau D De Guise JA Skalli W. Comparison of methods to assess quadriceps muscle volume using magnetic resonance imaging. J Magn Reson Imaging. (2009) 30:1116–23. 10.1002/jmri.21867 19856445

[8] Cruz-Jentoft AJ Bahat G Bauer J Boirie Y Bruyère O Cederholm T. Sarcopenia: revised European consensus on definition and diagnosis. Age Ageing. (2019) 48:16–31. 10.1093/ageing/afy169 30312372 PMC6322506

[9] Janssen I Heymsfield SB Wang Z Ross R. Skeletal muscle mass and distribution in 468 men and women aged 18–88 yr. J Appl Physiol. (2000) 89:81–8. 10.1152/jappl.2000.89.1.81 10904038

[10] Wilhelm EN Rech A Minozzo F Radaelli R Botton CE Pinto RS. Relationship between quadriceps femoris echo intensity, muscle power, and functional capacity of older men. Age. (2014) 36:1113–22. 10.1007/s11357-014-9625-4 24515898 PMC4082605

[11] Morse CI Thom JM Reeves ND Birch KM Narici MV. In vivo physiological cross-sectional area and specific force are reduced in the gastrocnemius of elderly men. J Appl Physiol. (2005) 99:1050–5. 10.1152/japplphysiol.01186.2004 15905324

[12] McPhee JS Cameron J Maden-Wilkinson T Piasecki M Yap MH Jones DA. The contributions of fiber atrophy, fiber loss, *in situ* specific force, and voluntary activation to weakness in sarcopenia. J Gerontol Ser A. (2018) 73:1287–94. 10.1093/gerona/gly040 29529132 PMC6132117

[13] Quinlan JI Franchi MV Gharahdaghi N Badiali F Francis S Hale A. Muscle and tendon adaptations to moderate load eccentric vs. concentric resistance exercise in young and older males. GeroScience. (2021) 43:1567–84. 10.1007/s11357-021-00396-0 34196903 PMC8492846

[14] Pons C Borotikar B Garetier M Burdin V Ben Salem D. Quantifying skeletal muscle volume and shape in humans using MRI: a systematic review of validity and reliability. PLoS ONE. (2018) 13:1–26. 10.1371/journal.pone.0207847 30496308 PMC6264864

[15] Shen W Wang Z Tang H Heshka S Punyanitya M Zhu S. Volume estimates by imaging methods: model comparisons with visible woman as the reference. Obes Res. (2003) 11:217–25. 10.1038/oby.2003.34 12582217 PMC1995086

[16] Tracy BL Ivey FM Metter EJ Fleg JL Siegel EL Hurley BF. A more efficient magnetic resonance imaging-based strategy for measuring quadriceps muscle volume. Med Sci Sports Exerc. (2003) 35:425–33. 10.1249/01.MSS.0000053722.53302.D6 12618571

[17] Barnouin Y Butler-Browne G Moraux A Reversat D Leroux G Béhin A. Comparison of different methods to estimate the volume of the quadriceps femoris muscles using MRI. J Med Imaging Heal Informat. (2015) 5:1201–7. 10.1166/jmihi.2015.150619856445

[18] Fouré A Nordez A Cornu C. Effects of eccentric training on mechanical properties of the plantar flexor muscle-tendon complex. J Appl Physiol. (2013) 114:523–37. 10.1152/japplphysiol.01313.2011 23239873

[19] McGlory C Gorissen SHM Kamal M Bahniwal R Hector AJ Baker SK. Omega-3 fatty acid supplementation attenuates skeletal muscle disuse atrophy during two weeks of unilateral leg immobilization in healthy young women. FASEB J. (2019) 33:4586–97. 10.1096/fj.201801857RRR 30629458

[20] Bland JM Altman DG. Statistical methods for assessing agreement between two methods of clinical measurement. Lancet. (1986) 1:307–10. 10.1016/S0140-6736(86)90837-8 2868172

[21] Layec G Venturelli M Jeong EK Richardson RS. The validity of anthropometric leg muscle volume estimation across a wide spectrum: from able-bodied adults to individuals with a spinal cord injury. J Appl Physiol. (2014) 116:1142–7. 10.1152/japplphysiol.01120.2013 24458749 PMC4097823

[22] Morse CI Degens H Jones DA. The validity of estimating quadriceps volume from single MRI cross-sections in young men. Eur J Appl Physiol. (2007) 100:267–74. 10.1007/s00421-007-0429-4 17342544

[23] Montano-Loza AJ Angulo P Meza-Junco J Prado CMM Sawyer MB Beaumont C. Sarcopenic obesity and myosteatosis are associated with higher mortality in patients with cirrhosis. J Cachexia Sarcopenia Muscle. (2016) 7:126–35. 10.1002/jcsm.12039 27493866 PMC4864157

[24] Cruz-Jentoft AJ Kiesswetter E Drey M Sieber CC. Nutrition, frailty, and sarcopenia. Aging Clin Exp Res. (2017) 29:43–8. 10.1007/s40520-016-0709-0 28155181

[25] GiovanniniSOnderGLattanzioFBustacchiniSdi StefanoGMoresiR. Selenium concentrations and mortality among community-dwelling older adults: results from ilSIRENTE study. J Nutr Heal Aging. (2018) 22:608–12. 10.1007/s12603-018-1021-929717761

[26] Lorenzi M Bonassi S Lorenzi T Giovannini S Bernabei R Onder G. A review of telomere length in sarcopenia and frailty. Biogerontology. (2018) 19:209–21. 10.1007/s10522-018-9749-5 29549539

[27] Lund H Christensen L Savnik A Boesen J Danneskiold-Samsøe B Bliddal H. Volume estimation of extensor muscles of the lower leg based on MR imaging. Eur Radiol. (2002) 12:2982–7. 10.1007/s00330-002-1334-1 12439580

[28] HajGhanbari B Hamarneh G Changizi N Ward AD Reid WD. MRI-Based 3D shape analysis of thigh muscles. Acad Radiol. (2011) 18:155–66. 10.1016/j.acra.2010.09.008 21111639

[29] Miokovic T Armbrecht G Felsenberg D Belavy DL. Heterogeneous atrophy occurs within individual lower limb muscles during 60 days of bed rest. J Appl Physiol. (2012) 113:1545–59. 10.1152/japplphysiol.00611.2012 22984243

[30] Smeuninx B Elhassan YS Manolopoulos KN Sapey E Rushton AB Edwards SJ. The effect of short-term exercise prehabilitation on skeletal muscle protein synthesis and atrophy during bed rest in older men. J Cachexia Sarcopenia Muscle. (2021) 12:52–69. 10.1002/jcsm.12661 33347733 PMC7890266

